# Resting state functional connectivity differences between behavioral variant frontotemporal dementia and Alzheimer's disease

**DOI:** 10.3389/fnhum.2015.00474

**Published:** 2015-09-08

**Authors:** Anne Hafkemeijer, Christiane Möller, Elise G. P. Dopper, Lize C. Jiskoot, Tijn M. Schouten, John C. van Swieten, Wiesje M. van der Flier, Hugo Vrenken, Yolande A. L. Pijnenburg, Frederik Barkhof, Philip Scheltens, Jeroen van der Grond, Serge A. R. B. Rombouts

**Affiliations:** ^1^Department of Methodology and Statistics, Institute of Psychology, Leiden UniversityLeiden, Netherlands; ^2^Department of Radiology, Leiden University Medical CenterLeiden, Netherlands; ^3^Leiden Institute for Brain and Cognition, Leiden UniversityLeiden, Netherlands; ^4^Alzheimer Center and Department of Neurology, VU University Medical CenterAmsterdam, Netherlands; ^5^Alzheimer Center and Department of Neurology, Erasmus Medical CenterRotterdam, Netherlands; ^6^Department of Neuropsychology, Erasmus Medical CenterRotterdam, Netherlands; ^7^Department of Clinical Genetics, VU University Medical CenterAmsterdam, Netherlands; ^8^Department of Epidemiology and Biostatistics, VU University Medical CenterAmsterdam, Netherlands; ^9^Department of Radiology and Nuclear Medicine, VU University Medical CenterAmsterdam, Netherlands; ^10^Department of Physics and Medical Technology, VU University Medical CenterAmsterdam, Netherlands

**Keywords:** Alzheimer's disease, frontotemporal dementia, functional connectivity, functional magnetic resonance imaging, neurodegenerative disorders, resting state fMRI, resting state networks

## Abstract

**Introduction:** Alzheimer's disease (AD) and behavioral variant frontotemporal dementia (bvFTD) are the most common types of early-onset dementia. Early differentiation between both types of dementia may be challenging due to heterogeneity and overlap of symptoms. Here, we apply resting state functional magnetic resonance imaging (fMRI) to study functional brain connectivity differences between AD and bvFTD.

**Methods:** We used resting state fMRI data of 31 AD patients, 25 bvFTD patients, and 29 controls from two centers specialized in dementia. We studied functional connectivity throughout the entire brain, applying two different analysis techniques, studying network-to-region and region-to-region connectivity. A general linear model approach was used to study group differences, while controlling for physiological noise, age, gender, study center, and regional gray matter volume.

**Results:** Given gray matter differences, we observed decreased network-to-region connectivity in bvFTD between (a) lateral visual cortical network and lateral occipital and cuneal cortex, and (b) auditory system network and angular gyrus. In AD, we found decreased network-to-region connectivity between the dorsal visual stream network and lateral occipital and parietal opercular cortex. Region-to-region connectivity was decreased in bvFTD between superior temporal gyrus and cuneal, supracalcarine, intracalcarine cortex, and lingual gyrus.

**Conclusion:** We showed that the pathophysiology of functional brain connectivity is different between AD and bvFTD. Our findings support the hypothesis that resting state fMRI shows disease-specific functional connectivity differences and is useful to elucidate the pathophysiology of AD and bvFTD. However, the group differences in functional connectivity are less abundant than has been shown in previous studies.

## Introduction

The most common types of early-onset dementia are Alzheimer's disease (AD) and behavioral variant frontotemporal dementia (bvFTD) (Ratnavalli et al., 2002). Patients with AD typically present with deficits in episodic and working memory (McKhann, 2011), whereas bvFTD is mainly characterized by changes in behavior, personality, and motivation (Rascovsky et al., 2011). However, symptoms may vary considerably, with overlap of symptoms between AD and bvFTD, including memory disturbances (Irish et al., 2014), and behavioral abnormalities (Woodward et al., 2010). Due to this heterogeneity and overlap of symptoms, clinical differentiation between both types of dementia may be challenging, particularly early in the disease. Therefore, to improve diagnostic accuracy and early differential diagnosis, there is a strong need for early markers of brain changes associated with the two types of dementia.

A substantial amount of dementia research used neuroimaging to elucidate the pathophysiology of bvFTD and AD (McMillan et al., 2014; Raamana et al., 2014). Neuroimaging of brain structure shows typical AD pathology in the hippocampus, precuneus, posterior cingulate cortex, parietal, and occipital brain regions (Buckner et al., 2005; Seeley et al., 2009; Krueger et al., 2010). BvFTD pathology is most often found in the anterior cingulate cortex, frontoinsula, and frontal brain regions (Seeley et al., 2009; Krueger et al., 2010).

Imaging of functional brain connectivity may be sensitive to detect disease-specific network changes in neurodegenerative diseases (Pievani et al., 2011). Former studies have shown abnormalities in functional connectivity in a posterior hippocampal-cingulo-temporal-parietal network known as the default mode network in AD (Greicius et al., 2004; Allen et al., 2007; Binnewijzend et al., 2012; Hafkemeijer et al., 2012) and in an anterior frontoinsular-cingulo-orbitofrontal network often called the salience network in bvFTD (Zhou et al., 2010; Agosta et al., 2013; Filippi et al., 2013; Rytty et al., 2013). Moreover, abnormalities in functional brain networks were found in mild cognitive impairment (Binnewijzend et al., 2012; He et al., 2014), subjective memory complaints (Hafkemeijer et al., 2013), and asymptomatic subjects at genetic risk for developing neurodegenerative diseases (Filippini et al., 2009; Sheline et al., 2010; Chhatwal et al., 2013; Dopper et al., 2014; Rytty et al., 2014), even in the absence of brain atrophy or cognitive decline.

Previous studies compared functional brain networks between dementia patients and controls, most often focusing on *a priori* defined regions or networks of interest, showing decreased functional connectivity in the default mode network in AD and in the salience network in bvFTD (Greicius et al., 2004; Allen et al., 2007; Binnewijzend et al., 2012; Agosta et al., 2013; Rytty et al., 2013). The direct comparison of functional connectivity between patients with AD and bvFTD, which is relevant for clinical differentiation, has been studied less often (Zhou et al., 2010; Filippi et al., 2013). In these studies, bvFTD patients have consistently shown decreased salience network connectivity compared with AD, while findings of default mode network connectivity have been inconsistent (Zhou et al., 2010; Filippi et al., 2013).

Therefore, to further explore functional connectivity in both types of dementia, the aim of this study was to compare whole-brain functional connectivity between AD and bvFTD. To study functional connections throughout the entire brain, voxel-based network-to-region (Greicius et al., 2004; Seeley et al., 2009; Zhou et al., 2010; Filippi et al., 2013) and region-to-region analyses (Supekar et al., 2008; Brier et al., 2014; Zhou et al., 2015) were applied. Given the differences in gray matter atrophy in AD and bvFTD (Buckner et al., 2005; Seeley et al., 2009; Krueger et al., 2010), we studied functional connectivity while controlling for gray matter volume. We expected connectivity differences in the posterior temporal-parietal regions of the brain in AD, and in the anterior cingulate and frontoinsular regions in bvFTD.

## Materials and methods

### Participants

We used resting state functional magnetic resonance imaging (fMRI) scans of 31 patients with probable AD, 25 patients with probable bvFTD, and 29 control participants (Table 1). All subjects were recruited from two Dutch centers specialized in dementia; the Alzheimer Center of the VU University Medical Center Amsterdam, and the Alzheimer Center of the Erasmus University Medical Center Rotterdam.

**Table 1:** **Characteristics of the study population**.

**Characteristic**	**HC (*n* = 29)**	**AD (*n* = 31)**	**bvFTD (*n* = 25)**	**AD vs. bvFTD (*p*-value)**
Age (years)	62.8 (5.1)	65.3 (7.0)	61.8 (7.3)	0.076
Gender (male/female)	17/12	19/12	19/6	0.249
Study center (VUMC/LUMC)^a^	16/13	20/11	16/9	0.969
Level of education^b^	5.4 (1.2)	4.9 (1.3)	5.1 (1.5)	0.580
Duration of symptoms (months)	n/a	41.9 (30.7)	49.4 (48.3)	0.355
MMSE (max score: 30)	28.8 (1.4)	22.7 (2.8)	24.4 (3.7)	0.068
FAB (max score: 18)	n/a	13.3 (3.4)	13.8 (2.8)	0.592
CDR (max score: 3)	0.0 (0.0)	0.8 (0.3)	0.7 (0.4)	0.545
GDS (max score: 15)	1.3 (1.5)	2.8 (2.9)	3.8 (3.3)	0.279
RAVLT immediate recall (max score: 15)	9.0 (2.3)	4.6 (1.6)	5.7 (1.8)	0.017
RAVLT delayed recall (max score: 15)	8.6 (3.2)	1.9 (1.8)	3.9 (3.5)	0.009
RAVLT total (max score: 75)	44.9 (11.3)	22.8 (8.2)	28.7 (9.1)	0.017
VAT (max score: 6)	5.9 (0.5)	3.3 (2.0)	4.8 (1.9)	<0.001
Digit span, forward (max score: 30)	12.1 (3.7)	10.0 (2.9)	10.6 (3.8)	0.519
Digit span, backward (max score: 30)	8.2 (3.4)	6.2 (2.7)	7.1 (3.1)	0.286
TMT A^c^	37.6 (14.0)	56.8 (32.3)	63.2 (52.0)	0.604
TMT B^c^	79.5 (26.8)	145.4 (65.3)	137.7 (67.5)	0.744
Stroop I^c^	46.6 (7.8)	56.1 (13.9)	58.7 (25.0)	0.637
Stroop II^c^	60.2 (9.6)	80.3 (31.4)	83.0 (41.6)	0.793
Stroop III^c^	98.3 (20.1)	156.5 (48.8)	153.1 (95.2)	0.883
Categorical fluency^d^	24.2 (5.4)	13.9 (5.1)	13.0 (4.7)	0.537
Letter fluency^d^	12.8 (5.1)	9.7 (4.0)	6.8 (4.3)	<0.001
LDST^d^	34.1 (6.8)	19.0 (9.3)	25.9 (7.1)	0.020

*AD, Alzheimer's disease; bvFTD, behavioral variant frontotemporal dementia; HC, healthy controls; MMSE, Mini-Mental State Examination; FAB, Frontal Assessment Battery; CDR, Clinical Dementia Rating Scale; GDS, Geriatric Depression Scale; RAVLT, Rey Auditory Verbal Learning Test; VAT, Visual Association Test; TMT, Trail Making Test; LDST, Letter Digit Substitution Test*.

*Values are means (standard deviation) for continuous variables or numbers for dichotomous variables. Scores on GDS were missing in 15 individuals, scores on TMT in 10, scores on Stroop in 1, scores on Categorical fluency in 6, scores on Letter fluency in 9, scores on LDST in 19, and scores on Digit span in 8 individuals*.

a *Imaging was performed either in the Alzheimer Center of the VU University Medical center (VUMC) or in the Leiden University Medical Center (LUMC) in the Netherlands*.

b *Level of education was determined on a Dutch 7-point scale ranging from 1 (less than elementary school) to 7 (university or technical college)*.

c *Time in seconds*.

d *Number of correct responses in 1 min*.

All patients underwent a standardized dementia screening including medical history, informant-based history, physical, and neurological examination, blood tests, extensive neuropsychological assessment, and magnetic resonance imaging (MRI) of the brain. Diagnoses were established in a multidisciplinary consensus meeting according to the core clinical criteria of the National Institute on Aging and the Alzheimer's Association workgroup for probable AD (McKhann, 2011) and according to the clinical diagnostic criteria for bvFTD (Rascovsky et al., 2011). To minimize center effects, all diagnoses were re-evaluated in a panel including clinicians from both Alzheimer centers.

The control participants were screened to exclude memory complaints, drug, or alcohol abuse, major psychiatric disorders, and neurological or cerebrovascular diseases. They underwent an assessment including medical history, physical examination, extensive neuropsychological tests, and an MRI of the brain, comparable to the work-up of patients.

Cognitive functioning of all participants was assessed using neuropsychological tests. The neuropsychological test battery included Mini Mental State Examination (MMSE) (Folstein et al., 1975), Frontal Assessment Battery (FAB) (Dubois et al., 2000), Clinical Dementia Rating scale (CDR) (Morris, 1993), Geriatric Depression Scale (GDS) (Reisberg et al., 1982), the Dutch version of the Rey Auditory Verbal Learning Test (Rey, 1958), Visual Association Test (Lindeboom et al., 2002), Wechsler Adult Intelligence Scale III subtest digit span (Wechsler, 1997), Trail Making Test part A and B (Army Test Battery, 1994), Stroop Color-Naming test (Stroop, 1935), Categorical and Letter fluency (Thurstone and Thurstone, 1962), and the Letter Digit Substitution Test (Jolles et al., 1995).

This study was performed in compliance with the Code of Ethics of the World Medical Association (Declaration of Helsinki). Ethical approval was obtained from the local ethics committees [VU University Medical Center Amsterdam (CWO-nr 11-04, METC-nr 2011/55) and Leiden University Medical Center (2011/55 P11.146)]. Written informed consent from all participants was obtained.

### Data acquisition

Imaging was performed on a 3 Tesla scanner either in the VU University Medical Center (Signa HDxt, GE Healthcare, Milwaukee, WI, USA) or in the Leiden University Medical Center (Achieva, Philips Medical Systems, Best, The Netherlands), using a standard 8-channel head coil.

For each subject, a three-dimensional T1-weighted anatomical image was acquired. Imaging parameters in the VU University Medical Center were: TR = 7.8 ms, TE = 3 ms, flip angle = 12°, 180 slices, resulting in a voxel size of 0.98 × 0.98 × 1.00 mm. Imaging parameters in the Leiden University Medical Center were: TR = 9.8 ms, TE = 4.6 ms, flip angle = 8°, 140 slices, resulting in a voxel size of 0.88 × 0.88 × 1.20 mm. In the Leiden University Medical Center an additional high-resolution echo planar imaging scan was acquired for registration purposes (TR = 2.2 s, TE = 30 ms, flip angle = 80°, 84 slices, resulting in a voxel size of 1.96 × 1.96 × 2.00 mm, including 10% interslice gap).

Resting state fMRI T2^*^-weighted scans were acquired using whole brain multislice gradient echo planar imaging. Imaging parameters in the VU University Medical Center were: TR = 1.8 s, TE = 35 ms, flip angle = 80°, 34 slices, resulting in a voxel size of 3.30 × 3.30 × 3.30 mm, including 10% interslice gap, 200 volumes, scan duration 6 min. Imaging parameters in the Leiden University Medical Center were: TR = 2.2 s, TE = 30 ms, flip angle = 80°, 38 slices, resulting in a voxel size of 2.75 × 2.75 × 2.99 mm, including 10% interslice gap, 200 volumes, scan duration 7 min and 33 s. Participants were instructed to lie still with their eyes closed and not to fall asleep during the resting state scan.

### Data analysis

Before analysis, all MRI scans were submitted to a visual quality control check to ensure that no gross artifacts were present in the data. Data analysis was performed with Functional Magnetic Resonance Imaging of the Brain Software Library (FSL 5.0.1, Oxford, United Kingdom) (Smith et al., 2004) and Matlab version R2011b (MathWorks, Natick, MA, USA). Anatomical regions were determined using the Harvard-Oxford cortical and subcortical structures atlas integrated in FSL.

#### Gray matter volume

Structural MRI scans were analyzed with a voxel-based morphometry (VBM) analysis (Ashburner and Friston, 2000) to study group differences in gray matter volume. First, the structural images were brain extracted and tissue-type segmented (Zhang et al., 2001). The resulting gray matter partial volume images were aligned to the gray matter MNI-152 standard space image (Montreal Neurological Institute, Montreal, QC, Canada) (Jenkinson et al., 2002), followed by non-linear registration (Andersson et al., 2007a). The images were averaged to create a study-specific template. Next, all native gray matter images were non-linearly registered to this study-specific gray matter template (Ashburner and Friston, 2000; Good et al., 2001). To correct for the contractions and enlargements due to the non-linear registration, each voxel of each registered gray matter image was multiplied by the Jacobian of the warp field, which defines the direction (larger or smaller) and the amount of modulation. The modulated segmented images were spatially smoothed with an isotropic Gaussian kernel with a full width at half maximum of 7 mm.

To study group differences in gray matter volume, a general linear model (GLM) approach using analysis of variance *F*-tests with *post-hoc* Bonferroni adjusted *t*-tests was applied. Age, gender, and study center were included as covariate in the statistical model. Voxel-wise non-parametric permutation testing (Nichols and Holmes, 2001) with 5000 permutations was performed using FSL-randomize correcting for multiple comparisons across space (statistical threshold set at *p* < 0.05, Family-Wise Error (FWE) corrected), using the Threshold-Free Cluster Enhancement (TFCE) technique (Smith and Nichols, 2009).

#### Preprocessing of resting state fMRI data

The preprocessing of the resting state data consisted of motion correction (Jenkinson et al., 2002), brain extraction, spatial smoothing using a Gaussian kernel with a full width at half maximum of 3 mm, and high-pass temporal filtering (cutoff frequency of 0.01 Hz). To quantify movement in the fMRI signal, the mean square of the absolute head movement was calculated. Patients with AD showed a mean square of 0.16 mm, patients with bvFTD 0.25 mm, and controls 0.19 mm. No significant group differences in movement values were found.

After preprocessing, the functional images were registered to the corresponding T1-weighted images using Boundary-Based Registration (Greve and Fischl, 2009). T1-weighted images were registered to the 2 mm isotropic MNI-152 standard space image (Montreal Neurological Institute, Montreal, QC, Canada) using non-linear registration (Andersson et al., 2007b) with a warp resolution of 10 mm. High-resolution echo planar images (only available for subjects scanned in the Leiden University Medical Center) were used for an additional registration step between functional images and T1-weighted images. In order to achieve better comparison across voxels, subjects, and centers, standardization on a voxel-by-voxel basis has been recommended (Yan et al., 2013). We used the Z-standardization approach in which individual resting state fMRI time series were normalized (standardized to z scores) on a voxel-by-voxel basis using the mean and standard deviation of each individual resting state signal across time (previously described in Yan et al., 2013).

Single-session independent component analysis was performed on the preprocessed resting state data to decompose the data into distinct components for denoising purposes (Beckmann and Smith, 2004). The standard training-dataset of FMRIB's ICA-based Xnoiseifier 1.05 (FIX) was used to auto-classify components into “good” (i.e., functional signal) and “bad” (i.e., noise) components (Salimi-Khorshidi et al., 2014). FIX removed unique variance related to “noise” components and motion confounds from the preprocessed fMRI data to denoise the resting state data and to increase the signal-to-noise ratio. Manual classification of data from 18 participants, equally distributed over groups and centers, showed that between 75 and 100% of the hand-labeled “noise” components and none of the “signal” components were removed.

#### Functional connectivity analysis: Network-to-region connectivity

Voxel-based group differences in network functional connectivity were studied using the dual regression method of FSL (previously described in Filippini et al., 2009). We used eight standard resting state networks as reference to study functional connectivity in a standardized way (Khalili-Mahani et al., 2012; Hafkemeijer et al., 2013). Resting state functional connectivity was determined in terms of similarity of the BOLD fluctuations in the brain in relation to characteristic fluctuations in the eight predefined resting state networks (Beckmann et al., 2005; Damoiseaux et al., 2006). These standardized resting state networks parcellate the brain into eight templates that represent over 80% of the total brain volume (Khalili-Mahani et al., 2012): network I) calcarine sulcus, precuneal cortex, and primary visual cortex (medial visual network), network II) superior and fusiform areas of lateral occipital cortex (lateral visual network), network III) superior temporal cortex, insular cortex, anterior cingulate cortex, auditory cortex, operculum, somatosensory cortices, thalamus (auditory system network), network IV) precentral and post-central somatosensory somatomotor areas (sensorimotor system network), network V) rostal medial prefrontal cortex, precuneal cortex, posterior cingulate cortex (default mode network), network VI) medial and inferior prefrontal cortex, anterior cingulate and paracingulate gyri, prefrontal cortex (executive control network), networks VII and VIII) frontal pole, dorsolateral prefrontal cortex, parietal lobule, paracingulate gyrus, posterior cingulate cortex (dorsal visual stream networks) (for further details, Beckmann et al., 2005; Khalili-Mahani et al., 2014). To account for noise, even after FIX, a white matter, and a cerebrospinal fluid template were included in the analysis (Fox et al., 2005; Birn, 2012).

In the dual regression, individual time series were first extracted for each template, using the eight resting state networks (Beckmann et al., 2005) and the two additional white matter and cerebrospinal fluid maps (Fox et al., 2005; Birn, 2012), in a spatial regression against the individual fMRI data set (regression 1). The resulting matrices described temporal dynamics for each template and individual. Next, the 10 temporal regressors were used to fit a linear model to the individual fMRI data set (regression 2), to estimate the spatial maps for each individual. This results in 10 3D images per individual, with for each voxel z scores representing the functional connectivity to each of the templates. The higher the absolute value of the z score, the stronger the connectivity to a network. Z scores were calculated for voxels both inside and outside the networks. Z scores of voxels outside a network indicate the strength of the functional connections between that outside region and the average of the network, while z scores of voxels inside the network indicate the strength of the connection between the average of the network and that region within the network. If functional connectivity is decreased in specific voxels inside the network, this indicates a less homogeneous fMRI signal in the network (i.e., less functional connectivity).

To study group differences in functional connectivity, a GLM approach using analysis of variance *F*-tests with *post-hoc* Bonferroni adjusted *t*-tests was applied. The data used in this study were collected from two centers. Although the distribution of participants between centers did not differ significantly between groups, we followed previous approaches to account for the potential effects of center and included center in all statistical models (Kim et al., 2009; Zhou et al., 2010). In addition to center, age, and gender were included as covariate. To account for potential effects of local structural gray matter differences within and between the two groups, segmented structural data were used to include gray matter volume of each voxel as subject-wise and voxel-wise covariates in the GLM design (Oakes et al., 2007). Voxel-wise non-parametric permutation testing (Nichols and Holmes, 2001) with 5000 permutations was performed using FSL-randomize correcting for multiple comparisons across voxels (statistical threshold set at *p* < 0.05, FWE-corrected), using the TFCE technique (Smith and Nichols, 2009).

#### Functional connectivity analysis: Region-to-region connectivity

In addition to the network analysis, we studied connections throughout the entire brain using correlation analyses. The whole brain was divided into 110 cortical and subcortical brain areas based on the probabilistic Harvard-Oxford cortical and subcortical structural atlas integrated in FSL (which we split into left and right hemisphere regions) to calculate the functional connectivity between pairs of anatomically defined brain areas. The preprocessed and denoised resting state data were voxel-based weighted with the gray matter partial volume estimate obtained with the FMRIB's Automated Segmentation Tool. Then, to calculate the average signal per probabilistic brain area, we averaged each voxel weighted by their probability per region (with a minimum of 25%) resulting in 110 time courses per subject. The full correlation between each pair of the 110 time signals was calculated, forming a weighted correlation matrix for each subject. Fischer-Z transformation was used to transform the correlations to z scores. The GLM approach using analysis of variance *F*-tests (IBM SPSS Statistics Version 20, IBM Corp., Somers, NY, USA) with *post-hoc* Bonferroni adjusted *t*-tests was applied to compare functional connectivity between pairs of anatomical regions in bvFTD and AD. The same statistical model as used in the network analysis was applied with age, gender, and study center included as covariates. To correct for multiple comparisons, we applied the false discovery rate (FDR) approach (*p* < 0.05) (Genovese et al., 2002). Group average of the control group was used as reference value in the *post-hoc* plots.

## Results

### Demographic characteristics

Demographic data for all participants are summarized in Table 1. There were no significant differences between the groups with regard to age, gender, study center distribution, level of education, and duration of symptoms (all *p* > 0.05). As expected, both dementia groups performed worse on cognitive tests compared with controls (all *p* < 0.05). Patients with AD performed worse compared with bvFTD patients on memory tests (Rey Auditory Verbal Learning Test, *p* = 0.009) and Visual Association Test, *p* < 0.001) and furthermore on the Letter Digit Substitution Test (*p* = 0.020). Patients with bvFTD performed worse compared with AD patients on Letter fluency (*p* < 0.001), which is an attention and executive function test. No significant differences between both dementia groups were found on the other cognitive tests (*p* > 0.05).

### Gray matter volume

**Figure 1:**
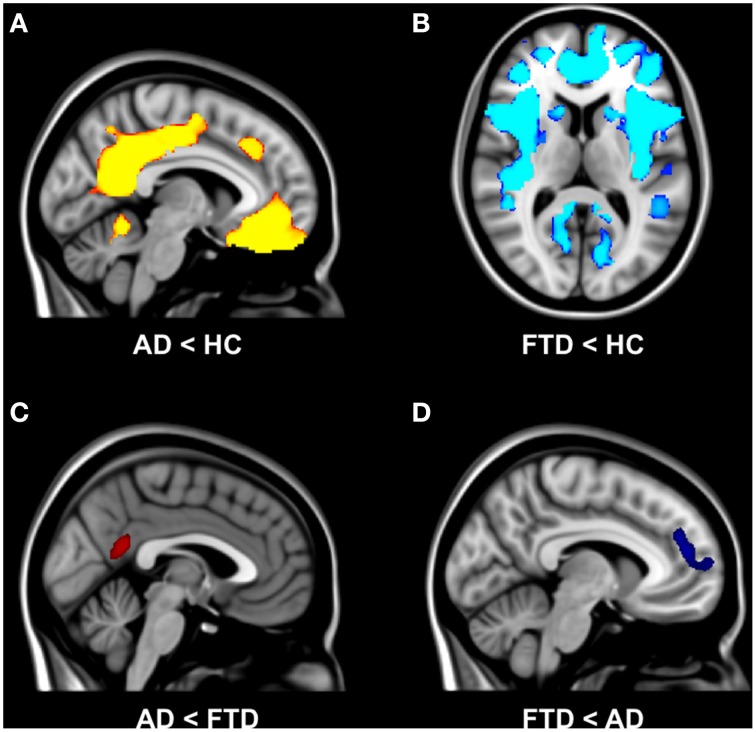
**Group differences in gray matter volume**. Differences in gray matter volume between behavioral variant frontotemporal dementia (FTD), Alzheimer's disease (AD), and healthy controls (HC) (TFCE, FWE-corrected). **(A)** Patients with AD showed less gray matter in precuneal cortex, posterior cingulate cortex, and frontal medial cortex compared with controls. **(B)** Patients with bvFTD showed less gray matter in anterior cingulate cortex, insular cortex, frontal pole, frontal gyrus, and middle temporal gyrus compared with controls. **(C)** AD patients had less gray matter compared with bvFTD in precuneal cortex and posterior cingulate cortex. **(D)** Patients with bvFTD had less gray matter compared with AD in anterior cingulate gyrus and frontal pole.

The voxel-wise structural analysis revealed group differences in gray matter volume (Figure 1). Patients with AD showed less gray matter in precuneal cortex, posterior cingulate cortex, frontal medial cortex, temporal gyrus, hippocampus, lateral occipital cortex, and operculum cortex compared with controls (Figure 1A). Patients with bvFTD showed less gray matter in anterior cingulate cortex, insular cortex, frontal pole, frontal gyrus, temporal pole, temporal gyrus, and temporal fusiform gyrus compared with controls (Figure 1B). AD patients had less gray matter compared with bvFTD in precuneal cortex, posterior cingulate cortex, and angular gyrus (Figure 1C). Patients with bvFTD had less gray matter compared with AD in anterior cingulate gyrus, frontal pole, and superior frontal gyrus (Figure 1D).

### Functional connectivity: Network-to-region connectivity

Functional connectivity analysis showed main effect of group for resting state network II, network III, network V, and network VIII. For the other four resting state networks, no group differences in functional connectivity were observed.

The results of *post-hoc* testing showed decreased functional connectivity in bvFTD compared with controls between network III (auditory network) and inferior temporal gyrus, middle temporal gyrus, superior temporal gyrus, post-central gyrus, and supramarginal gyrus (Table 2). In AD, functional connectivity was decreased compared with controls between network V (default mode network) and posterior cingulate gyrus, precuneal cortex, and lateral occipital cortex (Table 2).

**Table 2:** **Group differences in network functional connectivity**.

**Network**	**Brain structure^a^**	**Side**	**Peak voxel coordinates (MNI)**			**Peak *T*-score**
			**x**	**y**	**z**	
Default mode network AD < HC	Posterior cingulate gyrus	R	−2	−44	42	3.55
	Precuneal cortex	L	−16	−62	12	2.90
	Lateral occipital cortex	R	50	−74	26	3.74
Auditory system network bvFTD < HC	Inferior temporal gyrus	L	−42	−60	0	3.82
	Middle temporal gyrus	L	−52	−60	6	3.05
	Superior temporal gyrus	L	−60	−2	0	3.63
	Post−central gyrus	L	−50	−18	34	4.37
	Supramarginal gyrus	L	−52	−30	36	4.05
Lateral visual cortical network bvFTD < AD	Lateral occipital cortex/Angular gyrus	L	−40	−76	12	5.30
	Cuneal cortex	L	0	−86	32	4.39
	Cuneal cortex	R	22	−72	24	4.63
	Lateral occipital cortex	R	46	−78	10	3.86
Dorsal visual stream network AD < bvFTD	Lateral occipital cortex	L	−28	−72	52	5.05
	Parietal opercular cortex	L	−54	−24	24	4.36
	Lateral occipital cortex	L	−38	−70	22	4.18
Auditory system network bvFTD < AD	Angular gyrus	R	52	−62	44	5.22

*MNI, Montreal Neurological Institute, Montreal, QC, Canada; AD, Alzheimer's disease; HC, healthy controls; bvFTD, behavioral variant frontotemporal dementia; R, right; L, left*.

a *Full list of structures with group differences in network functional connectivity. Between group effects are independent of physiological noise, age, gender, study center, and gray matter volume. For each peak voxel x-, y-, and z-coordinates in the MNI-152 standard space image are given*.

**Figure 2:**
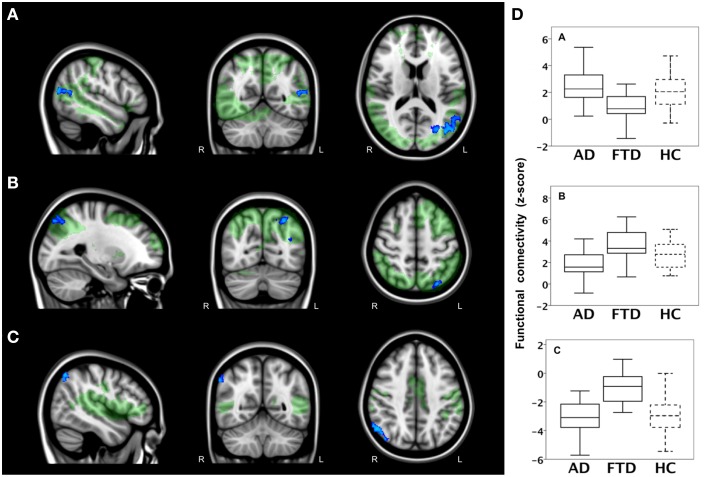
**Functional connectivity in bvFTD vs. AD: network-to-region connectivity**. Differences in functional connectivity between green networks and blue voxels in behavioral variant frontotemporal dementia (FTD) and Alzheimer's disease (AD) (TFCE, FWE-corrected). **(A)** Decreased functional connectivity between lateral visual cortical network (green) and lateral occipital cortex and cuneal cortex (blue) in bvFTD compared with AD. **(B)** Decreased functional connectivity between dorsal visual stream network (green) and lateral occipital cortex and parietal opercular cortex (blue) in AD compared with bvFTD. **(C)** Less negative functional connectivity between auditory system network (green) and angular gyrus (blue) in bvFTD compared with AD. Images are overlaid on the MNI-152 standard anatomical image. **(D)** Subjects' mean z scores were extracted from brain areas with group differences in functional connectivity (blue areas). Boxplots show median, lower, and upper quartile, and sample minimum and maximum z scores for patients with AD, patients with bvFTD, and healthy controls (HC, dotted lines).

The results of *post-hoc* testing showed decreased functional connectivity in bvFTD compared with AD between network II (lateral visual cortical network) and the lateral occipital cortex and cuneal cortex (Table 2, Figure 2A). Patients with AD showed decreased functional connectivity between network VIII (dorsal visual stream network) and lateral occipital cortex and parietal opercular cortex (Table 2, Figure 2B). We also observed “negative” (i.e., anti-correlated) connectivity: bvFTD patients showed less negative functional connectivity between network III (auditory system network) and the angular gyrus (Table 2, Figure 2C).

To illustrate group differences between both types of dementia and how network-to-region connectivity compares to controls, subjects' mean z scores of regions with differences in functional connectivity between the two patient groups are plotted in Figure 2D. Boxplots show mean z scores from: lateral occipital cortex and cuneal cortex (blue areas in A), lateral occipital and parietal opercular cortex (blue areas in B), and angular gyrus (blue areas in C).

### Functional connectivity: Region-to-region connectivity

**Figure 3:**
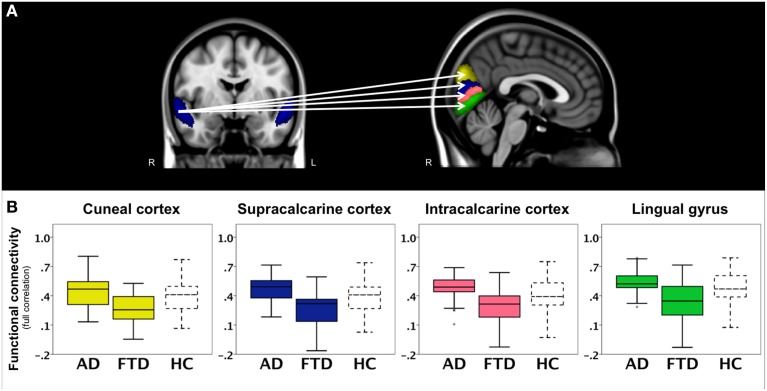
**Functional connectivity in bvFTD vs. AD: region-to-region connectivity**. Differences in pairwise functional connectivity between behavioral variant frontotemporal dementia (FTD) and Alzheimer's disease (AD). **(A)** Decreased functional connectivity between right superior temporal gyrus (blue area, coronal slice) and cuneal cortex (yellow), supracalcarine cortex (blue), intracalcarine cortex (pink), and lingual gyrus (green) in bvFTD compared with AD. Images show brain areas based on the probabilistic Harvard-Oxford structural atlas overlaid on coronal and sagittal slices of the MNI-152 standard anatomical image. **(B)** Subjects' correlation scores were extracted from brain areas with differences in functional connectivity between the two patient groups (right cuneal, supracalcarine, intracalcarine cortex, and lingual gyrus). Boxplots show median, lower, and upper quartile, and sample minimum and maximum correlation scores for patients with AD, patients with bvFTD, and healthy controls (HC, dotted lines).

In a second analysis, we studied connections throughout the entire brain using correlation analyses between 110 cortical and subcortical brain areas and found a main effect of group. The results of *post-hoc* testing showed decreased pairwise connectivity in bvFTD compared with AD between the right superior temporal gyrus (Figure 3A, blue area, coronal slice) and the right cuneal cortex (Figure 3A, yellow, *p* < 0.0001), left cuneal cortex (*p* = 0.0002), the right supracalcarine cortex (Figure 3A, blue, *p* = 0.0002), left supracalcarine cortex (*p* = 0.0001), the right intracalcarine cortex (Figure 3A, pink, *p* = 0.0001), and the right lingual gyrus (Figure 3A, green, *p* = 0.0002).

To further illustrate these group differences and how region-to-region connectivity compares to controls, functional connectivity with the right superior temporal gyrus is plotted in separate boxplots (Figure 3B) for the cuneal cortex (yellow), supracalcarine cortex (blue), intracalcarine cortex (pink), and lingual gyrus (green).

## Discussion

We studied functional connections throughout the brain in AD and bvFTD and showed that functional connectivity is different between both types of dementia. Given the gray matter atrophy, we observed decreased connectivity in bvFTD compared with AD between (a) the lateral visual cortical network and the lateral occipital cortex and cuneal cortex, and (b) between the auditory system network and the angular gyrus. Patients with AD showed decreased functional connectivity between the dorsal visual stream network and lateral occipital cortex and parietal opercular cortex. The decreased cuneal connectivity found in bvFTD patients was also found with the region-to-region connectivity analysis showing decreased connectivity between superior temporal gyrus and cuneal cortex, supracalcarine, intracalcarine cortex, and lingual gyrus. These findings support the hypothesis that resting state fMRI shows disease-specific functional connectivity differences and is useful to elucidate the pathophysiology of AD and bvFTD.

The region-to-region connectivity analysis showed decreased temporal gyrus connectivity in patients with bvFTD. This part of the brain is particularly vulnerable for FTD pathology, with gray matter atrophy located in this area (Whitwell et al., 2011; Farb et al., 2013). Decreased functional connectivity in the temporal lobe has earlier been found in bvFTD when compared with controls (Whitwell et al., 2011; Farb et al., 2013). In AD, we did not find differences in region-to-region connectivity, although decreased connectivity with the hippocampus has been reported by others (Allen et al., 2007).

The network-to-region analysis showed that functional connectivity of the angular gyrus was decreased (less negative) in patients with bvFTD. Differences in angular gyrus functional connectivity have been found in bvFTD compared with healthy controls (Farb et al., 2013; Rytty et al., 2013). An association between angular gyrus functional connectivity and stereotypical behavior has been found in bvFTD (Farb et al., 2013), suggesting an important role of the angular gyrus in the typical behavior of patients with bvFTD. However, the exact role of the angular gyrus in the behavior of bvFTD is not clear. Longitudinal changes in bvFTD have been found to be related to insular, not angular, connectivity (Day et al., 2013). Moreover, decreased angular functional connectivity was found in AD when compared with healthy controls (Wang et al., 2015) or with bvFTD patients (Zhou et al., 2010).

Functional connectivity between the dorsal visual stream network and the lateral occipital cortex and parietal opercular cortex is increased in bvFTD compared with AD. A recent study reported atrophy in the opercular cortex in patients with bvFTD, but no differences in functional connectivity in this brain area were reported in that study (Rytty et al., 2013). The opercular cortex overlies the insula, which is one of the brain regions first affected in bvFTD (Seeley et al., 2008). Patients with bvFTD show differences in insula functional connectivity compared with healthy controls (Farb et al., 2013) and patients with AD (Zhou et al., 2010). The differences in functional connectivity in this brain area may relate to the impaired social behavior that typically occurs in bvFTD, since the insula has an important role in social-emotional processing (Couto et al., 2013).

We found differences in functional connectivity in the so-called auditory system network that encompassed temporal cortex, insular cortex, anterior cingulate cortex, auditory cortex, operculum, somatosensory cortices, and thalamus, areas which are related to social-emotional processing (Seeley et al., 2007). We found decreased negative, not positive, functional connectivity between this network and the angular gyrus in patients with bvFTD. It has been suggested that negative functional connectivity indicates an anti-correlation between brain areas (Fox et al., 2005; Hampson et al., 2010). The interpretation of anti-correlations in resting state data is not straightforward (Fox et al., 2009; Yan et al., 2013) and its biological meaning is a subject of debate (Chai et al., 2012).

In addition to the direct comparison of AD and bvFTD patients, we compared functional connectivity between patients and control participants. As expected, we found decreased connectivity in AD compared with controls between the default mode network and posterior cingulate gyrus, precuneal cortex, and lateral occipital cortex. In bvFTD, functional connectivity was decreased compared with controls between the auditory network (temporal cortex, insular cortex, anterior cingulate cortex, auditory cortex, operculum, somatosensory cortices, thalamus) and temporal gyrus, supramarginal gyrus, and post-central gyrus. These findings reproduced connections that are comparable with other studies that compared functional brain networks between dementia patients and controls, showing decreased functional connectivity in the posterior temporal-parietal default mode network in AD and in the anterior cingulo-frontoinsular salience network in bvFTD (Greicius et al., 2004; Allen et al., 2007; Binnewijzend et al., 2012; Agosta et al., 2013; Rytty et al., 2013).

Decreased functional connectivity between regions within the default mode network has been found in AD compared with bvFTD (Zhou et al., 2010). The present study and the study by Filippi et al. (2013) were not able to replicate these default mode network disease-related differences. Although functional connectivity between patient and controls differed as expected, the group differences between AD and bvFTD were smaller than those in previous studies. This variability among studies may be due to multiple factors, including variations in the study cohort. Compared with other resting state fMRI studies in bvFTD and AD (Zhou et al., 2010; Filippi et al., 2013), we included a relatively large sample of patients in an early stage of the disease with very mild to mild symptoms. Hence it is possible that they are less severely affected compared with those in other studies. Furthermore, since early differential diagnosis between AD and bvFTD may be challenging, the possibility of misdiagnosis of the patients cannot be ruled out. The diagnosis FTD or AD can only be confirmed by brain autopsy after a person dies. In this study, postmortem data was not available. Nevertheless, all patients underwent an extensive dementia screening. Only dementia patients that fulfilled the most recent criteria for bvFTD (Rascovsky et al., 2011) and AD (McKhann, 2011) were included.

As expected, both dementia groups performed worse on cognitive functioning compared with controls. Patients with AD showed lowest scores on MMSE, which is a general measurement of cognitive performance (Folstein et al., 1975). Moreover, AD patients performed worse in memory functioning compared with controls and bvFTD. Patients with bvFTD performed, as expected, worse on executive functioning compared with controls. We further expected lower executive functioning scores in the bvFTD group compared with AD, however, AD patients did not differ from bvFTD in most executive functioning tests. Overall, the AD patients showed low scores in all cognitive domains, not only in the memory domain, but also in executive functioning. Although executive functioning could be useful to differentiate AD from bvFTD (Iavarone et al., 2004; Slachevsky et al., 2004), some studies reported that executive functioning, measured with FAB, does not discriminate AD from bvFTD patients (Lipton et al., 2005; Castiglioni et al., 2006). It has been suggested that testing multiple cognitive domains is required to differentiate both types of dementia rather than focus on one cognitive test (Lipton et al., 2005). In the current study, all patients underwent extensive neuropsychological assessment. Diagnoses were established according to the core clinical criteria for probable AD (McKhann, 2011) and for bvFTD (Rascovsky et al., 2011) and therefore were not based on one single neuropsychological test score.

This study showed data that were collected in two centers. The strengths of multicenter studies are the larger number of participants that can be included and the increased generalizability of the study findings. However, multicenter studies have also limitations, since the data will be less homogeneous than in single center studies. To increase homogeneity between centers in the current study, we evaluated all patients in a multidisciplinary panel including clinicians from different centers specialized in dementia, we used a standardization approach in order to achieve better comparison across voxels, subjects, and centers (Yan et al., 2013), and we added center as covariate in all statistical models, following previous approaches (Kim et al., 2009; Zhou et al., 2010).

## Conclusion

In the present study, we used resting state fMRI to study functional connections throughout the entire brain and showed that resting state functional brain connectivity is different between AD and bvFTD. Our findings support the hypothesis that resting state fMRI shows disease-specific functional connectivity differences and is useful to elucidate the pathophysiology of AD and bvFTD. However, the findings of the present study suggest that group differences in functional connectivity between both dementia types are less abundant than has been shown in previous studies.

## Author contributions

AH contributed to the design of the work, data acquisition, analysis, interpretation of the data, and drafting the manuscript. CM, ED, and LJ contributed to the data acquisition, interpretation of the data, and critically revised the manuscript. TS contributed to data analysis, interpretation of the data, and critically revised the manuscript. JS, WF, and HV contributed to the design of the work, data acquisition, interpretation of the data, and critically revised the manuscript. YP contributed to the data acquisition and critically revised the manuscript. FB and PS contributed to the design of the work and critically revised the manuscript. JG and SR contributed to the design of the work, data acquisition, analysis, interpretation of the data, and drafting the manuscript. All authors are accountable for all aspects of the work and approved the final version of the manuscript to be published.

### Conflict of interest statement

Anne Hafkemeijer, Christiane Möller, Elise G. P. Dopper, Lize C. Jiskoot, T. Schouten, John C. van Swieten, Yolande A. L. Pijnenburg, Jeroen van der Grond, and Serge A. R. B. Rombouts report no conflicts of interest. Wiesje M. van der Flier has received research support from Boehringer Ingelheim, Piramal Imaging, Roche BV, Janssen-Stellar, and speaker honoraria from Boehringer Ingelheim. All funds were paid to her institution. Hugo Vrenken has received research support from Merck-Serono, Novartis, and Pfizer, and speaker honoraria from Novartis. All funds were paid to his institution. Frederik Barkhof serves/has served on the advisory boards of: Bayer-Schering Pharma, Sanofi-Aventis, Biogen Idec, UCB, Merck-Serono, Novaritis, and Roche. He has been a speaker at symposia organized by the Serono Symposia Foundation. For all his activities he receives no personal compensation. Philip Scheltens serves/has served on the advisory boards of: Genentech, Novaritis, Roche, Danone, Nutricia, Baxter and Lundbeck. He has been a speaker at symposia organized by Lundbeck, Merz, Danone, Novartis, Roche, and Genentech. He serves on the editorial board of Alzheimer's Research and Therapy and Alzheimer's Disease and Associated Disorders. And he is a member of the scientific advisory board of the EU Joint Programming Initiative and the French National Plan Alzheimer. For all his activities he receives no personal compensation.

## Abbreviations

AD: Alzheimer's disease
bvFTD: behavioral variant Frontotemporal Dementia
CDR: Clinical Dementia Rating
FAB: Frontal Assessment Battery
FIX: FMRIB's ICA-based Xnoiseifier
fMRI: functional magnetic resonance imaging
FSL: Functional Magnetic Resonance Imaging of the Brain Software Library
FWE: Family-Wise Error
GDS: Geriatric Depression Scale
GLM: General Linear Model
MMSE: Mini-Mental State Examination
MNI: Montreal Neurological Institute
MRI: magnetic resonance imaging
TFCE: Threshold-Free Cluster Enhancement.
